# Perioperative Perfusion of Allografts with Anti-Human T-lymphocyte Globulin Does Not Improve Outcome Post Liver Transplantation—A Randomized Placebo-Controlled Trial

**DOI:** 10.3390/jcm10132816

**Published:** 2021-06-25

**Authors:** Paul Viktor Ritschl, Julia Günther, Lena Hofhansel, Stefanie Ernst, Susanne Ebner, Arne Sattler, Sascha Weiß, Annemarie Weissenbacher, Rupert Oberhuber, Benno Cardini, Robert Öllinger, Matthias Biebl, Christian Denecke, Christian Margreiter, Thomas Resch, Stefan Schneeberger, Manuel Maglione, Katja Kotsch, Johann Pratschke

**Affiliations:** 1 Department of Surgery, Campus Charité-Mitte and Campus Virchow-Klinikum, Charité—Universitätsmedizin Berlin, 13353 Berlin, Germany; paul.ritschl@charite.de (P.V.R.); sascha.weiss@klinikum-barnim.de (S.W.); robert.oellinger@charite.de (R.Ö.); matthias.biebl@charite.de (M.B.); christian.denecke@charite.de (C.D.); johann.pratschke@charite.de (J.P.); 2 Clinician Scientist Program, Berlin Institute of Health (BIH), Anna-Louisa-Karsch-Str. 2, 10178 Berlin, Germany; 3 Center for Operative Medicine, Department of Visceral, Transplant and Thoracic Surgery, Medical University of Innsbruck, 5020 Innsbruck, Austria; julia.k.guenther@gmail.com (J.G.); lhofhansel@ukaachen.de (L.H.); susanne.ebner@i-med.ac.at (S.E.); annemarie.weissenbacher@tirol-kliniken.at (A.W.); rupert.oberhuber@i-med.ac.at (R.O.); benno.cardini@i-med.ac.at (B.C.); christian.margreiter@i-med.ac.at (C.M.); t.resch@tirol-kliniken.at (T.R.); stefan.schneeberger@tirol-kliniken.at (S.S.); manuel.maglione@tirol-kliniken.at (M.M.); 4 Department of Psychiatry, Psychotherapy and Psychosomatics, Faculty of Medicine, RWTH Aachen, 52074 Aachen, Germany; 5 Biostatistics Unit, Clinical Research Unit, Berlin Institute of Health, Charité—Universitätsmedizin Berlin, 10117 Berlin, Germany; Stefanie.Ernst@PAREXEL.com; 6 Department of General, Visceral- and Vascular Surgery, Campus Benjamin Franklin, Charité—Universitätsmedizin Berlin, 12203 Berlin, Germany; arne.sattler@charite.de

**Keywords:** liver transplantation, ATLG, organ perfusion, organ pretreatment, machine perfusion, polyclonal antibody

## Abstract

Due to the lack of suitable organs transplant surgeons have to accept unfavorable extended criteria donor (ECD) organs. Recently, we demonstrated that the perfusion of kidney organs with anti-human T-lymphocyte globulin (ATLG) prior to transplantation ameliorates ischemia-reperfusion injury (IRI). Here, we report on the results of perioperative ATLG perfusion in a randomized, single-blinded, placebo-controlled, feasibility trial (RCT) involving 30 liver recipients (LTx). Organs were randomly assigned for perfusion with ATLG/Grafalon^®^ (AP) (*n* = 16) or saline only (control perfusion = CP) (*n* = 14) prior to implantation. The primary endpoint was defined as graft function reflected by aspartate transaminase (AST) values at day 7 post-transplantation (post-tx). With respect to the primary endpoint, no significant differences in AST levels were shown in the intervention group at day 7 (AP: 53.0 ± 21.3 mg/dL, CP: 59.7 ± 59.2 mg/dL, *p* = 0.686). Similarly, exploratory analysis of secondary clinical outcomes (e.g., patient survival) and treatment-specific adverse events revealed no differences between the study groups. Among liver transplant recipients, pre-operative organ perfusion with ATLG did not improve short-term outcomes, compared to those who received placebo perfusion. However, ATLG perfusion of liver grafts was proven to be a safe procedure without the occurrence of relevant adverse events.

## 1. Introduction

In times of changing demographics in regard to aging and increasing overweight and obesity, organ quality is a severe problem in the field of solid organ transplantation (SOT). Although organs of extended criteria donors (ECD) can be transplanted safely when appropriately selected and matched [1,2], these organs are more prone to ischemia-reperfusion injury (IRI) [2,3].

In the last decade, machine perfusion (MP) revolutionized the field of transplantation, resulting in improved organ viability and function [4,5,6,7,8,9]. Although there is no doubt about the beneficial effects of MP, a plethora of critical questions remain unresolved, including the duration of perfusion, the temperature and composition of the perfusate, surrogate parameters for organ quality, and possible strategies for recovery [10,11].

Although IRI is not yet fully understood, the detrimental effect on livers was shown to be associated with/conducted via the activation of Kupffer cells and neutrophils, the generation of reactive oxygen species (ROS), increased expression of adhesion molecules, and the production of cytokines and chemokines [12]. These factors promote the attachment of leukocytes to the endothelium and enhance the infiltration of immune cells [13]. Interestingly, T cells are attributed a particular role in the occurrence of IRI, although it represents an antigen-independent process [14,15]. Targeting IRI by applying various treatment schemes has been illustrated in an extensive number of experimental trials [16,17,18], but the translation of these experimental approaches into the clinic is still pending [19].

In the search for ready-to-use pretreatment regimens for marginal organs, both anti-human T-lymphocyte globulin (ATLG) and anti-thymocyte globulin (ATG) appeared to be a promising candidate as it has a long-standing history in various clinical applications, also in solid organ transplantation. Although commonly applied as an immunosuppressive agent, ATG successfully prevented IRI-related renal function impairment in rats when applied to the recipient [20]. The underlying mechanism can be explained by the downregulation of CCR7 expression on lymphocytes and the decreased response of monocytes to the released expression of CCL5 and CCL19, finally resulting in decreased chemotactic response [21]. In addition, it is anticipated that ATG induces regulatory mechanisms by expanding forkhead box P3 (FoxP3) positive T cells [22]. It is therefore not surprising that ATLG/ATG ameliorates IRI via different pathways [23,24,25]. In the setting of human liver transplantation, we could demonstrate that ATG has the potential to induce Tregs [26]. Besides experimental applications, ATG/ATLG is regularly used as an induction therapy method and to treat steroid-resistant rejection in the setting of SOT and—regardless of its potent immunosuppression—was proven to be a safe drug [27,28,29,30].

In a recently published RCT, we demonstrated that the perioperative perfusion of kidneys with ATLG prior to transplantation could significantly improve short-term outcomes [31]. Complementary to this successful trial, we performed a prospective, single-center, single-blinded, placebo-controlled RCT in livers to delineate whether perioperative ATLG-graft perfusion can ameliorate IRI also in the setting of LTx.

## 2. Materials and Methods

### 2.1. Trial Design

We performed a single-center, single-blinded (participants), parallel randomized, placebo-controlled trial at the Department of Visceral, Transplant and Thoracic Surgery, Medical University of Innsbruck, Austria. Patients were randomly assigned to the study groups in a 1:1 ratio using block randomization with varying block sizes and prepared randomization envelopes. The first patient underwent randomization on 8 August 2012 and the last on 20 June 2014.

For perfusion of livers, 25 mg of ATLG (Grafalon^®^, Neovii Biotech GmbH, Gräfelfing, Germany) was dissolved in 1000 mL saline and administered through the hepatic artery and portal vein (500 mL each) (ATLG perfusion = AP). Organs were perfused back-table after vessel preparation but prior to implantation. After 5–10 min of incubation, vessels were flushed with HTK (Custodiol ^®^, Dr. Franz Köhler Chemie GmbH, Deutschland) preservation solution to minimize systemic effects of “left-over” ATLG. Livers in the control group were placebo-perfused using saline only (control perfusion = CP). Biopsies were taken before and shortly after ATLG treatment and one hour after reperfusion. Immunosuppression was applied according to the center’s protocol. Liver transplant patients received no induction therapy. Maintenance therapy consisted of tacrolimus (initial dose of 6–10 ng/mL), methylprednisolone (500 mg with standardized tapering), and mycophenolate mofetil (1000–2000 mg per day).

### 2.2. Eligibility

Eligible patients were identified when a regular organ offer occurred to the patient listed for liver transplantation. Listing was prior indicated by an independent, interdisciplinary transplantation board. Only adult patients receiving a liver transplant from deceased donors were recruited. Diagnosis of HCV/HIV, as well as patients undergoing retransplantation or under a public guardian, were excluded.

### 2.3. Endpoints

Endpoints were defined similarly to our previously published trial [31]. The primary endpoint was defined as a change in graft function at day 7 from baseline, defined by the assessment of AST. In addition, graft function and patient survival were assessed correspondingly at other time points. Follow-up visits took place during the initial hospital stay (maximum day 15) and at 3, 6, and 12 months, when the study period ended. Furthermore, liver-transplant-related blood parameters as total bilirubin, alanine transaminase (ALT), gamma-glutamyl transpeptidase (GGT), alkaline phosphatase (ALP), and Quick value were measured. Complementary clinical factors including recipient and donor age, donor BMI, recipient age, recipient BMI, recipient sex, cold ischemia time (CIT), warm ischemia time (WIT), hospital stay, ICU stay and death/graft loss, and acute rejections were considered in the analysis.

### 2.4. Real-Time RT–PCR

Real-time reverse transcription–polymerase chain reaction (RT–PCR) was performed as recently described [31,32]. In brief, total RNA from snap-frozen biopsies was extracted using the NucleoSpin RNA Kit (Macherey-Nagel, Düren, Germany) according to the manufacturer’s instructions. The integrity of RNA was checked using a NanoDrop™ 2000c spectrophotometer. In the next step, cDNA was generated by reverse transcriptase reaction. The PCR for gene expression analysis was performed using a final volume of 25 µL containing 1 µL cDNA, 1 µL fluorogenic hybridization probe, 6 µL primer mix, 12.5 µL Master Mix (Life Technologies), and 5.5 µL distilled water. ABI PRISM 7500 Sequence Detection System (Life Technologies, Carlsbad, CA, USA) was used as a PCR cycler. Specific gene expression was normalized to the housekeeping gene hypoxanthine–guanine phosphoribosyltransferase (HPRT) using the formula 2-ΔCt [31].

### 2.5. Statistics

Statistical analysis was performed according to the previously published kidney transplantation trial [31]. A clinically relevant difference in graft function was estimated as a 50% decline of AST on day 7. The sample size was calculated assuming a 10% dropout rate with a statistical power of 85% and a two-sided significance level of 5%. An intention-to-treat analysis was performed with respect to the primary endpoint. No imputation for missing values for the primary analysis of longitudinal clinical parameters (percentage of change from baseline prior to transplantation) was undertaken since mixed models were applied for comparison adjusted for baseline. Kaplan–Meier charts in combination with log-rank testing were used to analyze overall patient and graft survival and to determine significance. Sensitivity adjustments were performed for age, recipient BMI, and recipient gender. No multiplicity adjustment was necessary. All analysis was carried out with appropriate parametric or nonparametric statistical tests based on their scale and distribution (Students’s *t* test/Mann–Whitney U test/X^2^). P values from nonprimary endpoints were considered in a nonconfirmatory, exploratory way. Testing for normal distribution was performed using histograms. PCR data were analyzed by applying a paired Mann–Whitney U test (testing between the study groups) or Kruskal–Wallis with Dunn post hoc test (testing within the group at the different time points of specimen retrieval). *p* values lower than 0.05 were considered statistically significant. SPSS (IBM V22.0) and GraphPad Prism 5 were utilized for data investigation.

### 2.6. Study Approval

This study was conducted according to the Declaration of Helsinki, in accordance with good clinical practice guidelines, and approved by an independent institutional ethics committee (ID: UN4640; date 23 July 2012). All participants provided written informed consent. The trial was registered on www.ClinicalTrials.gov (NCT03377283).

## 3. Results

### 3.1. Patients

**Figure 1:**
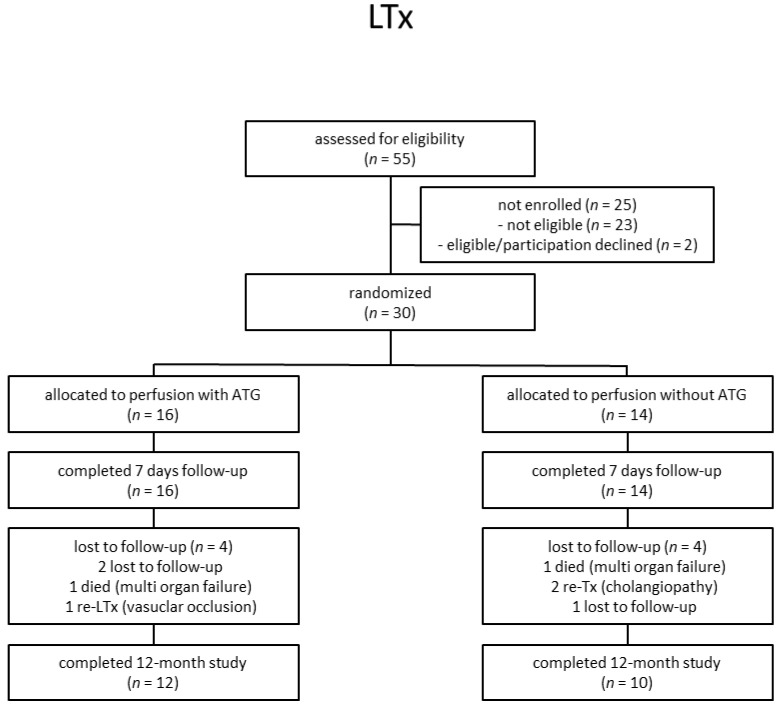
Participant flow diagram.

From 2012 to 2015, a total of 55 LTx recipients were assessed for eligibility (Figure 1). In total, 30 liver participants were eligible and enrolled in the study. Out of these 30 patients, 16 patients were allocated to receive a liver transplant perfused with ATLG, whereas 14 patients were randomly assigned to receive a liver perfused with saline. No significant differences regarding descriptive statistics were observed between ATLG and control patients (Table 1). In both groups, *n* = 4 patients were lost to follow-up, and *n* = 12 (AP) and *n* = 10 (CP) patients completed the observation period after 12 months (Figure 1). Duration of hospital stay was comparable between ATLG and control patients (Table 1). Surgical, biliary, infectious, and immunological complications were comparable, as summarized in Table 2. 

### 3.2. Liver Function after ATLG Perfusion

**Figure 2:**
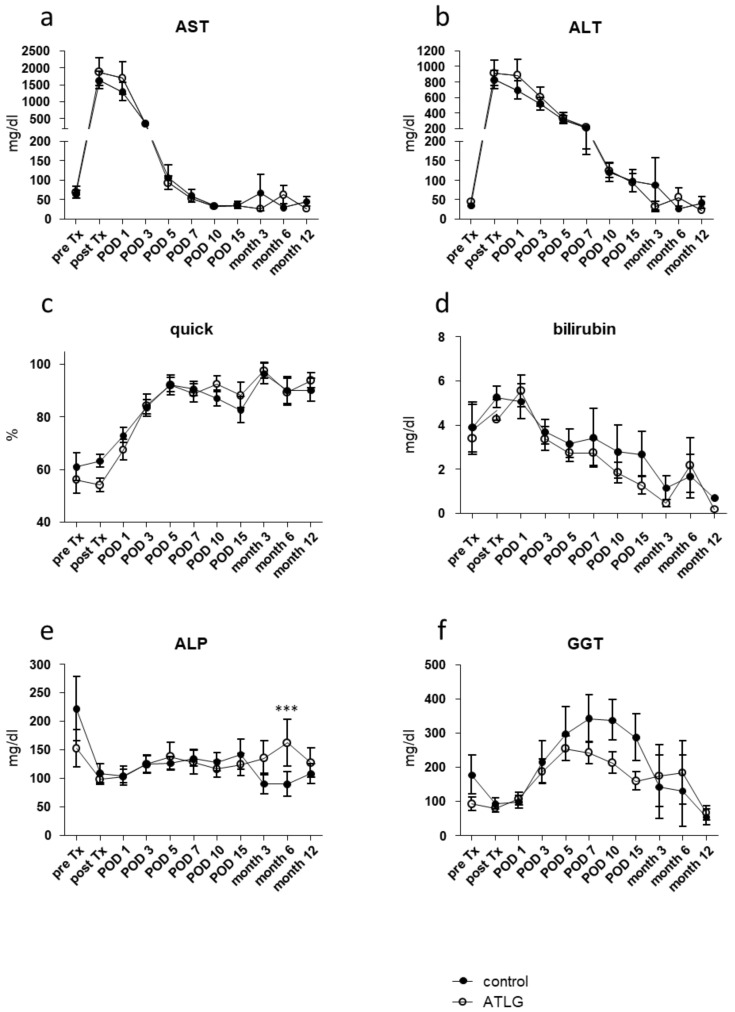
Functional liver parameter after liver transplantation. Results of routine blood analysis for the clinical parameters (**a**) alanine aminotransferase (ALT), (**b**) aspartate aminotransferase (AST), (**c**) Quick value, (**d**) bilirubin, (**e**) alkaline phosphatase, and (**f**) gamma-glutamyl transferase (GGT) are comparable for livers perioperatively perfused with ATLG as compared with control livers. ATLG: open circles; control: filled circles. data are presented as mean values ± SEM; *** *p* ≤ 0.001; ALP—alkaline phosphatase; ALT—alanine transaminase; AST—aspartate transaminase; ATLG—anti-human T-lymphocyte globulin; GGT—gamma-glutamyltransferase; POD—postoperative day.

In contrast to the kidney transplantation setting [31], both recipient groups receiving either an ATLG-perfused or a control-perfused organ displayed comparable clinical outcomes following LTx. Prior to transplantation, neither AST levels nor any other investigated liver parameter showed any difference between the groups (e.g., AST: AP-pre Tx: 68.1 ± 58.6 mg/dL versus CP-pre Tx: 63.6 ± 31.7 mg/dL) (Figure 2).

After a postoperative increase during the first two days post-transplantation, ALT and AST levels normalized over time until month 12. With respect to the primary outcome, no significantly different AST levels were detected between ATLG-perfused and control-perfused livers at day 7 (AST: AP-POD 7: 53.0 ± 21.3 mg/dL versus CP-POD 7: 59.7 ± 59.2 mg/dL, *p* = 0.686), and no further differences were observed for additional laboratory parameters, indicating graft function including Quick, total bilirubin, APT, and GGT (Figure 2).

### 3.3. ATLG Perfusion of Livers Does Not Influence the Inflammation Profile on mRNA Levels

**Figure 3:**
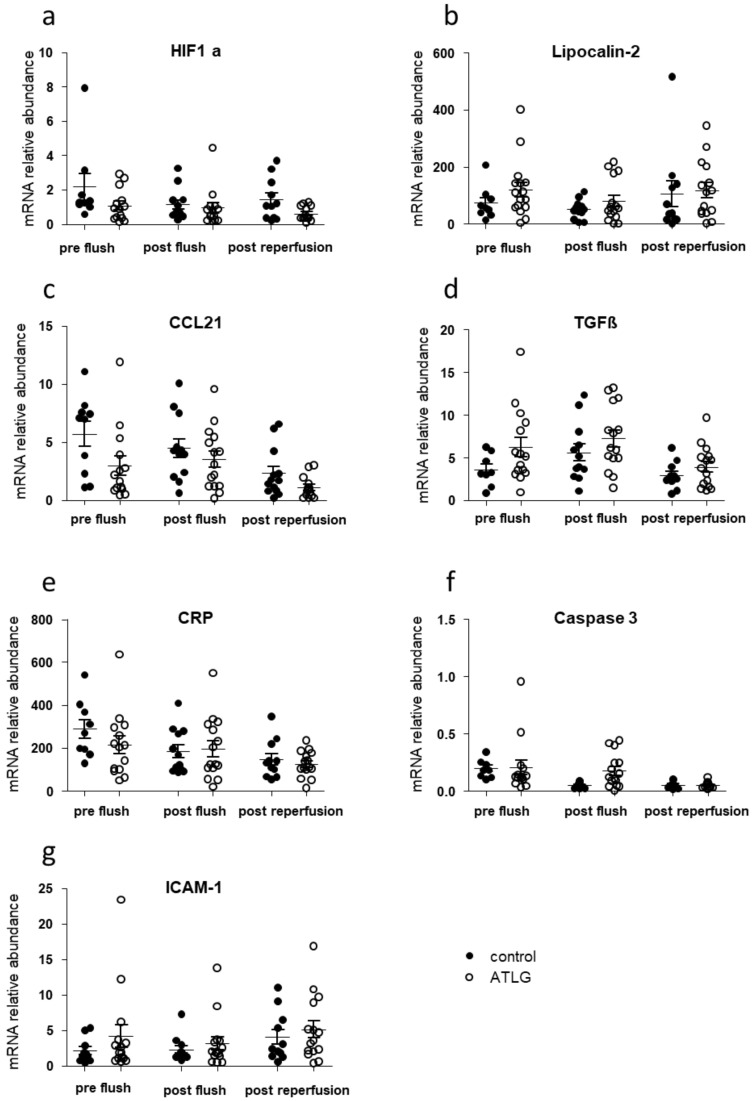
Inflammation-related candidate gene expression in liver biopsies taken before ATLG perfusion, after ATLG perfusion, and one hour post-reperfusion. Real-time quantitative PCRs of mRNA of whole tissues were performed at different time points perioperatively. Selected candidate markers (**a**) HIF1a, (**b**) Lipocalin-2, (**c**) CCL21, (**d**) TGFβ, (**e**) CRP, (**f**) Caspase 3, (**g**) ICAM-1), which play major roles in the context of inflammation, and cell adhesion did not show significant differences before perfusion with ATLG (pre-flush), directly after perfusion with ATLG (post-flush), or following reperfusion (post-reperfusion) between ATLG-perfused livers and control livers. ATLG: open circles; control: filled circles. Data are presented as mean ± SEM; ATLG—anti-human T-lymphocyte globulin; CCL21—chemokine (C-C motif) ligand 21; CRP—C-reactive protein; HIF1a—hypoxia-inducible factor 1-alpha; ICAM-1—intercellular adhesion molecule 1; TGFβ—transforming growth factor-beta.

We collected perioperative liver biopsies from transplanted organs at three different time points in order to monitor potential changes for inflammatory candidate markers as a consequence of ATLG perfusion. As displayed in Figure 3, we were not able to detect differences in the gene expression profiles for candidate genes indicative for inflammation, adhesion, and apoptosis between ATLG-perfused livers and controls at the different time points (Figure 3).

### 3.4. Patient Survival Rates Post-Transplantation

**Figure 4:**
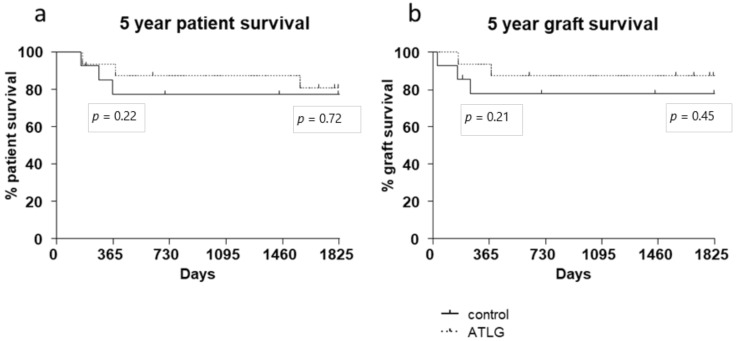
Patient and graft survival. No differences in (**a**) patient and (**b**) graft survival were detected between ATLG-perfused livers and control-perfused livers. The displayed p-values indicate 1- and 5-year survival. (AP: 93% 1-year patient survival vs. CP: 77% 1-year patient survival, *p* = 0.220; AP: 80% 5-year patient survival vs. CP: 77% 5-year patient survival, *p* = 0.718).

According to the assessed functional liver parameters and gene expression analysis, no differences in patient survival were detected for liver allograft recipients receiving an ATLG-perfused organ. A 1-year follow-up period showed no significant differences in patient survival (AP: 93% 1-year patient survival vs. CP: 77% 1-year patient survival, *p* = 0.220). After 5 years of follow-up, the survival curves were further approximated, and again no intervention-specific differences were observed (AP: 80% 5-year patient survival vs. CP: 77% 5-year patient survival, *p* = 0.718). Kaplan–Meier plots for graft survival demonstrate similar results (AP: 93% 1-year graft survival vs. CP: 77% 1-year graft survival, *p* = 0.214; AP: 87% 5-year graft survival vs. CP: 77% 5-year graft survival, *p* = 0.451) (Figure 4).

## 4. Discussion

Nowadays, liver transplantation is considered a standard procedure with excellent long-term outcomes. However, one of the limiting factors is the lack of appropriate organs [33]. Therefore, the donor pool is broadened steadily by accepting ECD organs and research is ongoing in order to improve these allografts [2,3]. For instance, the conditioning of brain-dead donors has been addressed only in a few prospective clinical trials so far and the effects of different therapeutic regimes including cortisol, dopamine, or hypothermia are limited [34,35,36,37,38]. On the other hand, conditioning of the organ by MP is a new way of organ preconditioning and shows promising results in a large number of clinical trials [5,6,8,10,39]. These studies mainly address the impact of organ perfusion versus cold storage, whereas alternative approaches for organ conditioning with substances other than oxygen/oxygen carrier are tested solely in preclinical models [40,41]. In contrast to machine perfusion with pure preservation solution, ATG mediates a plethora of biological effects ranging from directly targeting a variety of cell types (T cells, B cells, dendritic cells, and NK cells) but also modulation of cell adhesion, cell trafficking, and immune regulation [42].

Taken together, the perioperative application of ATG/ATLG to liver transplants was considered as an optimal strategy to precondition the organ in order to reduce IRI. In a recently published trial, we could demonstrate in a prospective RCT for the very first time that organ perfusion with ATLG during back-table preparation of the kidney graft results in reduced IRI [31]. In analogy to the kidney trial, we performed this liver-perfusion study, which is, to the best of our knowledge, the first time that liver organs were perfused with antibodies as a reconditioning strategy.

However, in contrast to the kidney trial, in which a significant reduction of creatinine (primary endpoint) and urea levels were observed in the early postoperative phase [31], no changes in short- or long-term outcomes were observed for the liver setting. Neither functional liver parameters nor patient or graft survival demonstrated any differences between the ATLG and control group (Figure 2 and Figure 4). Hence, with respect to the primary endpoint, the performed RCT must be seen as a negative trial.

Gene expression profiling of liver biopsies that were taken at three different time points during the process of transplantation did not reveal any differences in markers indicative of inflammation and cell adhesion (Figure 3). This finding was similar to the kidney transplantation setting, in which no differences in kidney graft biopsies were found 1 h after reperfusion [31]. This might be well explained by the fact that ATLG’s effect of binding to cellular structures is just unleashed after reperfusion. At this time, phagocytic cells, complement, and other parts of the immune system are flushed into the organ and start the biological processes [43,44]. Therefore, one hour post-reperfusion may be too early to see effects on gene expression. However, the collection of biopsies at a later time point were not ethically justified as complications might occur.

The observed differences between the two investigated organ systems remain unclear. It might be that the form of application used (back-table perfusion with 5–10 min incubation time) was in general too short. In this context, a continuous ATLG perfusion in combination with MP would be preferred and might result in different outcomes. Another alternative explanation for the detected interorgan differences might be the fact that liver and kidney, in general, react differently to immunological challenges, especially in the field of transplantation [32]. The underlying molecular and cellular mechanisms are diverse, but one of the main observations in this context is that liver grafts are more prone to tolerance, reflected by the fact that liver recipients need less immunosuppression [45,46,47,48,49].

Although performed as a prospective RCT, this study has several limitations. First, the patient cohort is relatively small. This might conceal smaller effects that would have been revealed in a large-scale RCT. Second, as mentioned above, the duration of organ perfusion and incubation with ATLG during back-table preparation might not be ideal for the effective binding of the polyclonal antibody. Nevertheless, ATLG might be an interesting pharmaceutical for reconditioning in a machine perfusion setting. Third, this study was performed as a single-center study, and therefore, location-specific peculiarities may limit the generalizability of the results. To overcome the mentioned limitations, several changes in future trial setups should be undertaken. Besides increased numbers of participants, the focus should be on marginal organs, as IRI treatment might be of greater relevance. In addition, the study should be performed in a multicenter setup including normothermic or oxygenated hypothermic machine perfusion.

## 5. Conclusions

In conclusion, this feasibility study demonstrates that the perioperative condition of liver organs with antibodies such as ATLG can be performed safely. Exploratory analysis showed no impact of ATLG perfusion on clinical parameters or on gene expression analysis of back-table biopsies. However, compared to a recently published kidney trial, the liver was once more shown to have unique immunological features in the context of organ transplantation and thus will require a specific setup for organ reconditioning.

## Figures and Tables

**Table 1:** Donor and recipient characteristics.

Variable	Total (*n* = 30)	ATLG-Perfused (*n* = 16)	Control-Perfused (*n* = 14)	*p*-Value ^#^
Recipient age (years ± SD) *	57 ± 10	55 ± 11	60 ± 8	0.113
Recipient gender (females)	5/30	4/16	1/14	0.336
Recipient BMI (kg/m^2^ ± SD) *	27 ± 5	25 ± 4	28 ± 5	0.078
MELD (+ IQR) **	16 (8)	17 (6)	15 (9)	0.355
Cold ischemic time (hours + IQR) **	9 (2)	9 (3)	9 (2)	0.24
Warm ischemic time (min + IQR) **	57 (16)	54 (19)	58 (16)	0.88
Disease ***				
Non-alcoholic steatohepatitis	14/30 (47%)	9/16 (56%)	5/14 (36%)	
Hepatocellular carcinoma	8/30 (27%)	4/16 (25%)	4/14 (29%)	
Cryptogenic cirrhosis	3/30 (10%)	2/16 (13%)	1/14 (7%)	
Primary biliary cirrhosis	2/30 (7%)	0/16 (0%)	2/14 (14%)	
Primary sclerosing cholangitis	1/30 (1%)	0/16 (0%)	1/14 (7%)	
Alcoholic liver disease	1/30 (1%)	0/16 (%)	1/14 (7%)	
Hepatitis B	1/30 (1%)	1/16 (6%)	0/14 (0%)	
Recipient ICU stay (days + IQR) **	5 (3)	4 (3)	5 (4)	0.481
Hospital stay (days + IQR) **	20 (8)	21 (10)	20 (9)	0.473
Death/ReTx (yes)	6/30	3/16	3/14	0.642
Donor age (years ± SD) *	55 ± 17	60 ± 13	51 ± 21	0.203
Donor BMI (kg/m^2^ ± SD) *	27 ± 5	26 ± 4	27 ± 5	0.69

* mean + standard deviation; ** median + interquartile range; *** count (percentage); ^#^ differences between ATLG and control; ATLG—anti-human T-lymphocyte globulin; ICU—intensive care unit; IQR—interquartile range; ReTx—retransplantation; SD—standard deviation.

**Table 2:** Adverse events.

Variable	Total (*n* = 30)	ATLG-Perfused (*n* = 16)	Control-Perfused (*n* = 14)	*p*-Value ^#^
Surgical events ***				
Anastomotic biliary strictures	3/30 (10%)	2/16 (13%)	1/14 (7%)	0.626
Bile leak	4/30 (13%)	2/16 (13%)	2/14 (14%)	0.886
Bleeding	2/30 (7%)	0/16 (0%)	2/14 (14%)	0.118
Infections ***				
CMV reactivation or infection	5/30 (17%)	3/16 (19%)	2/14 (14%)	0.743
Sepsis and MOF	4/30 (13%)	2/16 (13%)	2/14 (14%)	0.886
Aspergillom	1/30 (3%)	0/16 (0%)	1/14 (7%)	0.277
Intracerebral abscess	1/30 (3%)	0/16 (0%)	1/14 (7%)	0.277
Others ***				
Rejection	4/30 (10%)	1/16 (6%)	3/14 (21%)	0.222
Non-anastomotic biliary strictures	3/30 (10%)	3/16 (19%)	0/14 (0%)	0.088
Other bilary complications	1/30 (3%)	1/16 (6%)	0/14 (0%)	0.341

*** count (percentage); some patients had >1 event; ^#^ differences between ATLG and control; ATLG—anti-human T-lymphocyte globulin; MOF—multiple organ failure.

## Data Availability

The data presented in this study are available on request from the corresponding author.
